# Service user involvement for mental health system strengthening in India: a qualitative study

**DOI:** 10.1186/s12888-016-0981-8

**Published:** 2016-07-28

**Authors:** Sandesh Samudre, Rahul Shidhaye, Shalini Ahuja, Sharmishtha Nanda, Azaz Khan, Sara Evans-Lacko, Charlotte Hanlon

**Affiliations:** 1 Center for Chronic Conditions and Injuries, The Public Health Foundation of India, Plot No. 47, Sector 44, Institutional Area Gurgaon, Delhi NCR 122002 India; 2 Institute of Psychiatry, Psychology and Neuroscience, Health Services and Population Research Department, King’s College London, PO29 David Goldberg Centre, De Crespigny Park, London, SE5 8AF UK; 3 London School of Economics and Political Science, Personal Social Services Research Unit, Houghton Street, London, WC2A 2AE UK; 4 Department of Psychiatry, Addis Ababa University, College of Health Sciences, School of Medicine, 6th Floor, College of Health Sciences Building, Tikur Anbessa Hospital, PO 9086 Addis Ababa, Ethiopia; 5 The Public Health Foundation of India House No. 19, Rishi Nagar Char Imli, Bhopal, 462016 India

## Abstract

**Background:**

There is a wide recognition that involvement of service users and their caregivers in health system policy and planning processes can strengthen health systems; however, most evidence and experience has come from high-income countries. This study aimed to explore baseline experiences, barriers and facilitators to service user-caregiver involvement in the emerging mental health system in India, and stakeholders’ perspectives on how greater involvement could be achieved.

**Methods:**

A qualitative study was conducted in Sehore district of Madhya Pradesh, India. In-depth interviews (*n* = 27) and a focus group discussion were conducted among service users, caregivers and their representatives at district, state and national levels and policy makers, service providers and mental health researchers. The topic guide explored the baseline situation in India, barriers and facilitators to service user and caregiver involvement in the following aspects of mental health systems: policy-making and planning, service development, monitoring and quality control, as well as research. Framework analysis was employed.

**Results:**

Respondents spoke of the limited involvement of service users and caregivers in the current Indian mental health system. The major reported barriers to this involvement were (1) unmet treatment and economic needs arising from low access to mental health services coupled with the high burden of illness, (2) pervasive stigmatising attitudes operating at the level of service user, caregiver, community, healthcare provider and healthcare administrators, and (3) entrenched power differentials between service providers and service users.

Respondents prioritised greater involvement of service users in the planning of their own individual-level mental health care before considering involvement at the mental health system level. A stepwise progression was endorsed, starting from needs assessment, through empowerment and organization of service users and caregivers, leading finally to meaningful involvement.

**Conclusions:**

Societal and system level barriers need to be addressed in order to facilitate the involvement of service users and caregivers to strengthen the Indian mental health system. Shifting from a largely ‘provider-centric’ to a more ‘user-centric’ model of mental health care may be a fundamental first step to sustainable user involvement at the system level.

## Background

The Government of India introduced the National Mental Health Policy in October 2014 outlining the prioritized agenda for extending, within a pragmatic time-frame, basic mental health care facilities to all sections of the population across the country by 2020. The tactical vehicle for implementing the policy is the National Mental Health Program, launched in 1983, that was further revised in 2011.

India was one of the first low and middle income countries (LAMICs) to launch a national mental health program with the aim of extending community based mental health care through the existing primary health care system [9]. Although progress has been made in shifting from institution based care to a system of primary mental health care; implementation challenges have meant that locally available service provision is uneven. The consequence is a large treatment gap, with only about ten per cent of those with mental health problems able to access effective care [22]. In addition, there is an increasing burden of mental health disorders in India [27]. This unmet need for mental health care carries a high burden in terms of suffering, disability, stigma and discrimination, unemployment, premature mortality and risk of human right abuses [19, 35].

Over the last four decades, many countries have implemented interventions to increase service user and caregiver (SU-CG) involvement in various aspects of health system strengthening, specifically involvement in policy making, planning, service delivery, service monitoring and evaluation, training, advocacy and research. In the mental health system, service user is a person with mental illness who has availed any kind of mental health care and caregiver is a person primarily responsible for caring of a person with mental illness [21]. Although there is a lack of conceptual clarity around the term [8], service user and caregiver involvement has been described by Tritter et al., as a “way in which patients and their families will be able to draw on their experience and apply their priorities to the development, organization, delivery and evaluation of the mental health services” [40]. In this schema, involvement is defined as direct or indirect, individual or collective and proactive or reactive [40]. Historically, mental healthcare has implemented ‘indirect involvement’, whereby decision makers in the system invite service users and their caregivers to generate information, but the decision to act on the information is retained by the decision makers [40]. Furthermore, mental healthcare has tended to focus more on the individual involvement rather than collective involvement and the nature of involvement has been essentially reactive with less sustainability.

In scaling up access to mental health care, SU-CG involvement has the potential to make health systems more responsive to the needs of the people it is meant to serve and may benefit patients directly by increasing the likelihood of recovery [14]. Involvement may be particularly important given the social marginalization and vulnerability of mental health service users coupled with the low priority given to mental health care within the health system [26, 31]. The global evidence suggests that SU-CG involvement amends the mental health system perspective to become more accountable and responsive towards people with mental illness [38]. Service users and their caregivers are the only people that are present throughout the continuum of care and thus, if engaged and empowered, they would be able to play a significant role in ensuring a positive health experience and to provide insights into system challenges and how these may be overcome [6, 13].

The health system has been described as “a dynamo of shifting sub systems and interacting synergies”. A framework for action to strengthen health systems has been proposed, conceptualising the health system ‘building blocks’ (governance, service delivery etc) that together constitute a complete system in the manner of multiple relationships and interactions among the blocks- one affecting and influencing the other” [6]. This framework emphasizes that when service users and the community are placed in the center as driving actors, the system strengthens due to enhanced equity, social justice, participation and inter-sectoral collaboration. With higher levels of service user involvement, sustainable system strengthening is more likely to be attained.

Sound empirical evidence on the outcomes of SU-CG involvement activities remains underdeveloped in many LAMICs, including India, and information is lacking on how to translate the globally acquired knowledge into practice [3]. For example, Indian health system has Rogi Kalyan Samiti (RKS) as a community mobilization and participation initiative [29]. RKS is a patient welfare committee facilitating community members to participate and ensure the proper management and functioning of the public healthcare facilities. RKS although accounts for the perspective of community and service users; do not provide evidence or strategies for effective SU-CG involvement [12].

In order to make meaningful SU-CG involvement a reality in India, there is a pressing need to understand the existing level of SU-CG involvement, the barriers to and facilitators for involvement, and potentially acceptable and feasible approaches to enhance involvement in the future. Elucidating the pathways and strategies towards SU-CG involvement in the mental health system has the potential to address inequality in the Indian health system more broadly and pave the way for service user involvement in relation to a range of public health priorities in India, including communicable and non-communicable diseases, injuries and reproductive and child health.

The Emerald (Emerging Mental Health Systems in Low and Middle Income Countries) program is a multi-country study investigating key aspects of mental health system strengthening in LAMICs [33]. Developing the evidence and experience base for SU-CG involvement in mental health system strengthening is a priority for Emerald as a cross-cutting issue. Therefore, as part of the Emerald program, this qualitative study aims to explore the existing level of SU-CG involvement in the Indian mental health system and investigate the barriers and facilitators to the greater involvement to strengthen the system. This study explores range of stakeholders’ perspective in the Indian mental health system to explore and inform the planning of interventions to promote greater SU-CG involvement.

To this purpose, we addressed the following research questions:

1. What is the current experience of people with mental illness (and/or their main caregivers), representatives from the public health system and former patients representing caregiver organizations in terms of involvement in mental health policy making, planning, service development, monitoring, research and evaluation in India?
2. What are the barriers and facilitators to service users and caregivers’ involvement in mental health system level activities in India?

## Methods

A qualitative study using in-depth interviews and a focus group discussion was conducted amongst key informants from February to March 2014.

### Study participants and setting

#### Policy makers-planners

The participants in the policy-makers/planners group were selected from the ‘National Mental Health Policy group’ (NMHP) [25]. The Government of India’s Ministry of Health and Family Welfare constituted the NMHP group in May 2011. The key objective of the NMHP group was to prepare a National Mental Health Care Plan with specific reference to the National Mental Health Program and the District Mental Health Program (DMHP) and to outline specific strategies and activities to implement the priority areas of action identified in the National Mental Health Care Policy. The members of the NMHP group included service users, mental health researchers, psychiatrists, public health experts and policy makers representing national level health system [25]. Among 12 members of the NMHP group, we selected six participants using purposive sampling and conducted the interviews. The distribution of six participants from NMHP group is listed in a Table for distribution of six participants in policy makers-planners group: [Table 1: characteristics of the participants in policy makers-planners group]

**Table 1:** Characteristics of the participants in policy makers-planners group

Policy makers and planners (*n* = 6)	Participants
Senior Psychiatrists	2
Service user	1
Representative of user organization	2
Mental health researcher	1

For the policy-makers/planners group at state level, we selected officials from the State ministry of health, Government of Madhya Pradesh (MP). The second largest state in India; Madhya Pradesh is situated in the central part of India and has a population of 72.5 million which accounts for 6 % of the India’s total population [5]. This study was nested in the Emerald and PRIME programs that are implemented in MP [36]. In Madhya Pradesh, the DMHP is operational in the Sehore district, where the mental health program has been functioning through collaboration between the DMHP and the PRogram for Improving Mental health carE (PRIME) since 2011 [10, 20].

#### Service providers

We selected public health service providers working in MP at the state and district level. At district level, the service providers were medical officers providing general outpatient public health services and mental health services through the PRIME-DMHP program support. This group also consisted of a DMHP psychiatrist and psychologist in Sehore district.

#### Representatives of user-caregiver organizations

For this group, we selected organizations on the basis of their strong representation in the NMHP group [25]. The respondents in this group were service users and caregivers with strong social representation in the Indian mental health domain.

#### Service users and caregivers

For this group, we selected participants from the Sehore district site. In Madhya Pradesh, DMHP is operational in the Sehore district, where the mental health program has been functioning through collaboration between the DMHP and PRIME since 2011 [20]. Therefore, we selected service users registered at the public healthcare facilities in Sehore district.

Focus group discussion (FGD): we conducted one FGD with the representatives of users and caregiver in the Sehore district. There were 15 participants in the FGD. The participants included two service users, three caregivers, eight community members and two community advisors for the DMHP in the Sehore district.

### Sample

A total of 27 in-depth interviews (IDIs) were conducted: eight with service users, three with caregivers and 16 with policy makers, planners, service providers and service user and caregiver representatives. The socio demographic characteristics of participants are illustrated in [Table 2: Socio-demographic characteristics of participants]

**Table 2:** Socio-demographic characteristics of participants

Participant information	Policy makers and planners (*n* = 6)	Service providers (*n* = 7)	Representatives from user and caregiver organizations (*n* = 3)	Service users and caregivers in Sehore District (*n* = 11)
Age in years Mean (Range)	51 (35–66)	37 (33–46)	41 (38–48)	33 (22–47)
Gender				
Male	4	3	3	7
Female	2	4	0	4
Mean education level	Graduate	Graduate	Graduate	Seventh grade

### Topic guide

The themes covered in the interview schedule were derived from the experiential knowledge of the study team. The interview schedule focused on SU-CG involvement in policy making, planning, service development, quality monitoring and research in relation to the mental health system. Experiences of SU-CG involvement and barriers and facilitators for greater involvement were explored. The questions were not read out verbatim and emphasis was given to understand the perspective of the respondents. Pilot interviews with two representatives were carried out to assess the relevance of the topic, timing, feasibility and research burden. Based on the feedback from the respondents during pilot interview and discussion amongst the researchers, the interview schedule was modified.

### Data collection procedure

The interviews were audio taped and field notes were taken after obtaining the participant’s informed consent. All interviewers were trained in qualitative research methodologies and had prior experience in conducting a qualitative research. Interviewers were public health professionals with good understanding of the Indian mental health system. Interviews were conducted in the local Hindi language, were transcribed verbatim in Hindi and then translated into English. We back translated selected transcripts.

We presented the preliminary findings from the study at the India International Public Health Conference in November 2014 and at the Public Health Foundation of India Research Symposium in March 2015. In both the meetings, feedback was centered on the need to include more information on the suggested intervention pathway for greater SU-CG involvement and to discuss the stigma gap that was perceived as an overarching barrier at all levels of the system. We further revised the results and discussion section based on the feedback.

### Data analysis

During the initial stages of tool development for the study, the provisional plan for data analysis was structured. The transcripts were analysed using framework of thematic content analysis with the assistance of a qualitative software package NVivo9 [23]. While identifying themes, an effort was made to understand convergence and divergence of views and how contextual factors could affect the similarity and difference in views. As new themes emerged, they were also included in the thematic content analysis. The preliminary analysis was presented to experienced academics and clinicians to assess the plausibility of the findings.

## Results

Many of the respondents had difficulty speaking about current service user and caregiver (SU-CG) involvement in the mental health system given extremely low levels of existing involvement. The respondents discussed the gaps in the system that they perceived to be the main reason behind the lack of SU-CG involvement in the mental health system.

Respondent (R): “Zero! I mean barring small pilots done by NGOs (non-government organization), within the public sector there is absolutely no service user or caregiver involvement in mental health system. Although in NHM (National Health Mission), there are some initiatives such as RKS (Rogi Kalyan Samiti) and VHS (Village Health Society) that have community accountability system in place, mental health system at the moment has no improvement when it comes to involvement of service user and caregiver.”

ID01: Member of the national mental health policy group

Barriers to greater involvement included unmet basic needs of service users (for example, access to treatment, functional recovery and livelihoods), pervasive stigma operating at multiple levels, and power differentials between service providers and SU-CG. Overcoming barriers to involvement was seen as a process, requiring a stepwise progression from assessing and meeting unmet needs, followed by empowerment and mobilization of service users, before meaningful involvement could be achieved. Greater involvement of service users in their own mental health care was prioritised by service users and their caregivers over collective mental health system strengthening activities.

### Basic need- related factors

Service users spoke about their unmet needs due to inadequacies of the existing Indian mental health system. As a consequence, their capacity to contribute actively within society was thought to be constrained.

Respondents emphasized the need for reliable access to quality mental health care. The ‘*treatment gap’* arising from the lack of accessible mental health services was considered to be a fundamental barrier to greater involvement of mental health service users in mental health system strengthening activities. Respondents noted that service users and their families need basic mental health services first; involvement comes as a much later priority. When compared with other health conditions, respondents noted that mental health services are given low priority and therefore availability and accessibility of mental health services is limited.

“R: in India, it is difficult to involve service user and caregivers in mental health system for strengthening activities. Majority of the states do not know the mechanism and effects of service user involvement. Availability of basic mental health service is a priority for service users in India today. Today limited numbers of psychiatrists see approximately 300 patients every day in outpatient services and they are concentrated in urban areas. There has to be a better treatment roll out first for service user involvement.”

In-depth interview number 06 (ID06): Member of national mental health policy group

In the absence of accessible and affordable public mental health services, respondents discussed the issue of care and livelihood among service users. Often service users and their families end up spending considerable amounts of money on accessing mental health services from private health sector providers. This financial burden from help-seeking is exacerbated by the reduction in income associated with the disability associated with prolonged mental illness.

“R: in this hospital (district hospital), I have been coming for last 20 years. I have many times received medicines for fever and infection. But they (general physicians) don’t give me medicines for my mental illness (psychosis). I have to wait for specialist (psychiatrist) to come and give me medicines once a month or two. If my medicines are finished early, I have to travel to nearby Bhopal city to meet private psychiatrist and spend about two thousand rupees (`) for one visit. So money is big problem for me when it comes to my disease. Without public health services, it is difficult for me to treat my disease and get well for my children’s sake.”

ID19: Service user availing services in Sehore District Mental Health Program and PRIME

As with access to quality mental health care, the quest for livelihoods was major issue amongst service user and was afforded greater priority than involvement in service and system strengthening.

Quote: “R: Service development term is intimidating in a sense that service users don’t get adequate services. Their quest for livelihood is major issue in addition to stigma and discrimination. In this context for service users, priority is to get their condition cured. Then only will they be able to earn, marry and have family. Therefore, involvement in service development is not present priority for service users and even caregivers.”

ID14: user survivor representing a national level service user organization

### Stigma

Respondents spoke of the negative impact of stigma against people with mental health illness. Stigma was reported to operate as a barrier to SU-CG involvement not only at all levels of the health system, including the mental health system, but also within the community and even amongst service users themselves. Indeed, the effect of stigma was perceived to be one of the most important obstacles to greater SU-CG involvement.

Perceived stigma among service users:

“R: A service user does not like to be identified with other service users. Every service user feels his/her problem is different than other service users and hence needs different attention. Therefore, a service user does not wish to get organized in a group of mentally ill due to many reasons including stigma and fear being exposed in community as mentally ill. Also this revelation has certain negative consequences like loss of employment and community rejection.”

ID14: Recovered service user representing a national level service user organization

Respondents perceived the stigma of being a ‘service user’ to be stronger in urban settings resulting in less willingness to accept that they had a mental illness and seek treatment due to fear of social ostracization. Indeed, urban stigma was thought to outweigh the fact that an urban user might be better informed about mental illness and mental health care, as well as having greater resources to be able to seek out and pay for care.

Quote: “R: in India, many service user and caregiver organizations work on awareness and service delivery section. After recovery, some service users remain associated with the organization providing voluntary contribution in treatment and recovery of other patients. But many service users refuse to get involved in awareness and policy making or planning activities, especially in urban settings. The reason is, for these activities service user has to come out in open and disclose his or her former illness to society. Due to fear of social annihilation, most of the service users refuse to get involved in activities on higher platform.”

ID14: service user representing a national level service user organization

Stigma towards people with mental illness operating at the level of the health facility (doctors, nurses, health workers) was felt to obstruct service users from having any say in service delivery and quality monitoring.

Quote: “R: Service users are mentally ill, hence are not capable or even trained to make right decisions for services provided to them. Hence even after recovery, they are not eligible to monitor the services provided to them.”

ID13: Medical officer in (service provider) working in district level health care facility

Stigmatising attitudes towards involvement of service users were also evident in relation to mental health policy and planning activities, as exemplified in the following comment:

“R: service user involvement is okay in grass root direct service delivery components such as monitoring and research. But service users or caregivers should not be involved in policy making for mental health. They are not capable of making informed decisions at higher level such as policy making and planning. Policy making is very much higher decision making attribute and hence only high official policy makers should make decisions related to policy making for mental health.’

ID05: Senior officer in State Health Department

### Power differentials in the health system

Respondents spoke of entrenched power differentials within the Indian health education system and public health system that serve as an obstacle to the involvement of service users and caregivers in health system strengthening process. A description of a provider-centric system emerged, with little emphasis on holistic approaches to care, therefore precluding any notion of SU-CG involvement.

Quote 1: “R: Disjunction between clinicians and social scientists exists at various levels of the mental health system. While building capacity of service users; behavioural and attitudinal change of professionals (even community) towards respecting service user involvement is also essential.”

ID02: member of national mental health policy group

Quote 2: “The Indian medical education system including psychiatry does not provide any kind of orientation towards patient-centric care or service user involvement. In addition, the system enables medical doctors to be the statuary decision makers in the health systems interventions and service delivery. This way the present medical education system enables health system to become provider-centric.

ID01: member of national mental health policy group

Service providers were perceived as considering all service users to be weak and ignorant component in the system and hence incapable of contributing to system in any way.

“R: in our country hierarchy is respected by both the parties (professional service providers and service users). It needs some effort to change this mind set. Professionals (service providers) don’t like Service user or caregiver participation. They don’t consider them capable of such participation.”

ID14: Recovered service user representing a national level service user organization

When discussed whether service users should be involved in activites like ‘anti-stigma program’ that is implemented by the District Mental Health Program (DMHP), service providers summarily rejected the notion of involvement.

R: No. They (service users) are illiterate. They would not understand these activites. We (service providers) should be consulted. We know more. Also we can involve front line workers and other paramedic workers to a limited extent only. Ultimately we (service providers) are going to treat a person with mental illness.”

ID07: Medical officer (service provider) working in district level health care facility

### Steps to involvement

**Fig. 1:**
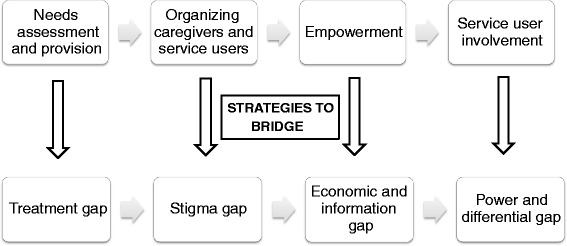
A stepped model for intervention explaining service user involvement pathway bridging the mental health system gaps

While mentioning the gaps and barriers to SU-CG involvement, respondents also suggested strategies to bridge these gaps to facilitate effective and sustainable SU-CG involvement in the mental health system. The consensus among all respondents was that SU-CG involvement cannot be achieved in the absence of certain pre-requisite conducive factors. Involvement was rather conceptualized as a higher step that is achieved only after ensuring certain steps such as fulfilment of basic needs and empowerment of user and caregivers. Drawing on the respondent perspectives, a stepped model for intervention is presented in [Fig. 1: a stepped model for intervention explaining service user involvement pathway bridging the mental health system gaps].

Our findings indicate a need for a stepwise action model to achieve sustainable SU-CG involvement. It suggests that involvement is not a onetime activity but a dynamic process to bridge the aforementioned gaps.

### Service user needs assessment

Quote: “R: I came across a mother of a child with a developmental disability. Although medical professional was trying to improve child’s condition through various medicines and therapy, the mother’s need was to have child call her ‘mother’ first. This significant instance illustrates situation of mental health care in our system. Often in mental health services, service users need basic services with adequate accessibility, affordability and emotional and financial support for prolonged care; that is often required among mentally ill way before involvement.”

ID02: member of national mental health policy group

Respondents noted that people with mental illness have unfulfilled basic needs that require immediate attention in the system. These needs were noted as basic mental health services and community support. Fulfilling these needs is a first step for a sustainable SU-CG involvement that consequently bridges the treatment gap in the system.

### Organizing service users

As reported during the Focus Group Discussion (FGD), a service user may be reluctant to associate with other service users and share his/her problem in a group and disclose it out in the open. Prioritising the caregivers” involvement as a first step was thought to be an acceptable and feasible way to open the door for future involvement of service users.

Quote from FGD: “R: mental health service users due to many reasons including self-perceived stigma do not express a wish to get involved in health system domains. Caregivers however due to less stigma and first-hand experience of mentally ill in family, are more willing and capable for involvement in the system. Service user and caregiver organizations observe that caregiver involvement in mental health system can work as an opening door for service user involvement. The implication is that caregivers are indeed persons observing mental health related stigma and sufferings at first hand basis. Caregivers are also stigmatized but certainly less than service users. Caregiver led groups are more sustainable due to their own commitment to care for their kin with mental illness and deep understanding towards stigma and discrimination”

FGD1: Community members representing service user organizations at district and state level mental health system.

### Support group

Quote from FGD “R: we have a patient group here. Patients’ support group and caregivers’ support group are also different. They should fall in different category only…. They should not fall into a single category. Second thing is that this support group should be facilitated by someone. A leader’s presence is required there. Because if patient only shares his viewpoint he will start giving his share of complains only. Our first experience was such that to bring them together in a group itself is a great thing. All the patients coming together… So later on, after a year, we realized that it has now became a complaint group only…. Because they start to share that whose problem is more vigorous. We had to lead the group towards the recovery. How our attitudes can change? How do we view the situations differently and then direct our efforts is important. This must have had a definite impact on the group. A facilitator is needed for that. And the facilitator should know all these things like till where and in which direction he has to take this group. That is how every time it happens… We, our trainees have brought in 5-6 different places (for such groups). So we started it with the group of caregivers because that is relatively easy.

FGD1: Community members representing service user organizations at District level

### Empowerment

Empowering service users was considered to be a critical step to address the stigma and economic gap in the existing system. Service users and caregivers are often socially marginalized and socio-economically disadvantaged. Ensuring financial incentive, community support and meritorial recognition for involvement can empower service users and their caregivers.

Quote “R: Persons with mental illness are many times, unaware of their surrounding and are not capable of involvement without our (health worker) support and training. We can train and support recovered service users in our community and increase awareness among them regarding their basic rights in mental health system. These service users should be provided with some basic incentives for their active involvement as often they are very poor and unemployed.”

ID12: service provider (community health worker) working at District level health centre.

### Involvement

Quote: “R: involvement is a crucial step. Service users often face huge level of stigma during their illness and even after recovery. Therefore, most of the times after recovery, service users do not talk about their formal mental illness. This is turning point, because getting involved in the mental health system means disclosing his or her mental illness in society in spite of possible stigma and discrimination. Incentives such as meritorial recognition, stipend and publicity act as positive factors for service user involvement.”

ID14: Recovered service user representing a national level service user organization

In the last step, respondents considered that incentives in various forms could operate as motivating factors to ensure successful service user involvement. Sustained involvement of service users and caregivers was expected to progressively diminish the unwillingness of stakeholders to work with service users.

## Discussion

Historically, mental health services in India have been inequitably distributed and the accessibility of quality care has been a major challenge; especially in rural and tribal regions. With our study, we explore two major domains: what is the level of service user involvement in the current mental health system and how increased SU-CG involvement might strengthen the system, particularly with respect to service development and delivery, quality monitoring and advocacy.

In order to understand perspective of people with mental illness availing mental health services, we recruited service users and caregivers form Sehore, a rural district in Madhya Pradesh, a central state in India. since 2011 in Sehore District, PRIME RPC and District Mental Health program is providing integrated mental health care in a primary health care service platform [36]. Therefore, we recruited mental health service users and caregiver availing public mental health services in Sehore district to understand their experience of SU-CG involvement.

The results from our study reaffirm that there is a very little involvement of service users and caregivers and the major reported barrier is the lack of accessible quality mental health care. Rather than being a reason not to attempt greater service user involvement, we feel that this lack of basic quality services underlines the need for SU-CG involvement in the service delivery block. Over the past three decades of a national mental health program, in the absence of or with little user involvement, the services have remained poor in quality and inaccessible [11]. Therefore, a ‘bottom up’ approach of user involvement is needed in order to attain sustainable improvement in mental health care and increase accessibility for services. Involvement of users and caregivers is pertinent at each level of service delivery to ensure accessible and quality mental health care.

The barriers identified to SU-CG involvement in the Indian context are consistent with those from many LAMICs [14]. In the Indian health system, mental health is not a policy priority. This low priority has led to inadequate access to mental health care in the country.

Another barrier observed in our study is that the existing mental health system is ‘provider-centric’. In the Indian health system paradigm, medical professionals (doctors, nurses, health workers) play central role in the system and the system does not function without them [37]. Provider-centric health systems have been highlighted previously as an important barrier to service user involvement [39]. The traditional model of medical education does not prepare professionals to involve service users in the system. As a result, health workers’ attitudes towards service users in general, and people with mental illness in particular deems them as incompetent and incapable of decisions or choices. This situation results in a provider-centric health system that precludes any service user and caregiver involvement.

Incorporating a more holistic model of care into medical education could help broaden service providers’ perspectives. SU-CG involvement also addresses a key issue of trained human resources in the Indian mental health system. Service user and caregiver involvement has a potential to provide a sensitive human resource that can amend the system to be more responsive and accountable to the needs of those it meant to serve (people with mental illness). This process can help to tackle the scarcity of human resources in the Indian mental health system and mental health systems in other LAMICs as well [32].

Pervasive stigma towards people with mental illness has been observed historically as a significant barrier to SU-CG involvement across mental health system blocks, be it policy making, service development or research [19]. Improving public attitudes can create a virtuous cycle and increase empowerment among service users [7]. However, respondents in this study emphasise that stigma and social marginalization currently prevent service users from getting actively involved in any component of the system. This could be considered as a ‘stigma gap’. Stigma is a potent negative force that operates at multiple levels. Societal stigma [4] towards people with mental illness can establish a negative social context for people with mental illness and their families [18, 28]. Provider level stigma [16] towards service users may impede access and engagement with services and treatment and the recovery pathway [1]. Service users hence may internalise negative stereotypes and therefore interventions to address self-stigma are needed so that service users do not become further disempowered [4, 17]. Thus, in order to facilitate service user involvement, stigma needs be tackled at multiple levels in society, health care systems and services and among service users [30, 34]. Our study participants indicated that self-stigma and fear of social exclusion was even stronger in urban settings among people with mental illness, indicating that stigma is pervasive despite relatively better education and awareness levels. Therefore, stigma needs to be tackled at each level in the building blocks of the mental health system and needs to be addressed distinctly in its own right [6].

### Strategies to increase SU-CG involvement

India’s National Mental Health Program is set for major changes in the wake of the new national mental health policy bill [15]. At this opportune time, our study attempts to investigate potential strategies for enhanced service user involvement in the Indian mental health system to address basic needs of service users, stigma and power differentials within the system that restrict SU-CG involvement [24].

This study echoes the global evidence that there is a lack of clarity on what involvement specifically means [8]. In line with the empirical evidence, this study suggests that involvement shall be seen as a process that starts with individual service user and caregiver involvement and then moves towards the collective involvement strengthening all the building blocks of the mental health system [40]. This study supports the need for proactive involvement rather than historical models of reactive SU-CG involvement that resulted in weak sustainability.

The study suggests that empowering service users and caregivers through: capacity building by community workers, meritorial recognition in the community and incentive-based support will help overcome self-stigma. One approach has been tried in other LAMICs is to organise service users through a chain of self-help groups [2].

Caregivers led self-help groups (SHG) have been found to motivate service users for organized efforts towards recovery. Once organized, service users can discuss common problems faced by them in the mental health system and would then try to address these problems on various platforms in a systematic manner. Effective information dissemination among caregivers and service users can be envisaged as a powerful tool in this process [2]. Optimal service user involvement is only possible if all of the above mentioned steps are properly implemented.

Barriers to service user involvement and potential strategies for enhanced involvement are two countering factors. Implementing the potential strategies will help overcome various barriers to SU-CG involvement in the Indian mental health system. Given the three decades old national mental health program, there is no paucity of experience among various stakeholders related to barriers to SU-CG involvement in the system. There appears to be common ground among all stakeholders in the mental health system in terms of their willingness to tackle the barriers for greater SU-CG involvement.

We considered that it was important to include a broad range of stakeholders to ensure all aspects of the phenomenon under study were covered. The inclusion of different stakeholders such as policy makers, service providers, researcher and service users-caregivers allowed us to triangulate the findings and look for any preliminary evidence of differing perspectives. Future work on SU-CG involvement could look in greater depth at specific aspects of health system strengthening or from the perspective of a particular participant group.

### Limitations and scope of the study

One of the limitations for this study is that we interviewed people with mental illness availing services in Sehore District in Madhya Pradesh (India) through collaborative program of DMHP and PRIME and their caregivers only. Another limitation of the study is the small number of caregivers included (*n* = 3), which limited out ability to draw out differences of perspectives between service users and caregivers.

## Conclusion

Strong barriers such as a provider-centric health system, self-stigma, negative attitudes among service providers and stigma present in the mental health system currently prevent service user and caregiver involvement. A more ‘holistic approach’ and multi system caregiver led strategies are needed for greater service user involvement. There is a need to change the system from ‘provider-centric’ to ‘user-centric’ for strengthening the mental health system in India.

## Abbreviations

DMHP, District Mental Health Program; Emerald, Emerging mental health systems in low and middle Income countries; FGD, focus group discussion; IDI, in-depth interview; LAMIC, low and middle income country; NGO, non-government organisation; MP, Madhya Pradesh; NHM, National Health Mission; NMHP, National Mental Health Policy; PRIME, programme for improving mental health care; R, respondent; RKS, Rogi Kalyan Samiti; SHG, self-help group; SU-CG, service user and caregiver; VHS, Village Health Society
